# Mr. Guthrie's Case of Hydrocephalus

**Published:** 1800-09

**Authors:** T. Guthrie

**Affiliations:** Berwic


### To Dr. DYCE, Aberdeen.

Dear Sir,

I Have obferved in the courfe of my reading and practice, that Hydrocephalus Internus has always been attended with fatal confequences. It is, therefore, in my opinion, the duty of every practitioner to contribute as much as is in his power to the elucidation of a difeafe fo fatal to the human race. I fubmit to your confideration the following cafe, which lately came under my care, and which differs confiderably from all others that I have yet met with; if you think that it deferves a place in the Medical and Phyfical Journal, I beg leave to requeft the favour of you to tranfmit it to the Conductors of that valuable publication.

About the end of May laft, I was called to fee a child about feven weeks old; I found him very emaciated and weak; his head much enlarged, and the futures confiderably feparated. From thefe fymptoms, I was led to conclude, that there was water in his head. His appearance and fituation feemed to indicate that he could live only a few days; I therefore did not attempt any cure, but was induced to enquire particularly what ftate he was in prior to my feeing him. I was informed, that he was a ftout healthy child when he was a month old, and that his head had no uncommon appearance. He took fevere fits of crying every afternoon for the four firft weeks of his age, which was attributed to gripes: he generally made lefs water than is ufual for children at his time of life, and always foamed at the mouth when afleep. In general, he refted very ill; but flept better in the forenoon than at any other time. He had a good appetite; his food was flour bread and milk, but he vomited whenever he took bread and water: he was always regular and eafy in his bowels. When a month old, he was feized with a fever, which lafted two weeks, and during its continuance, he cried almoft day and night. At the commencement of the fever his head began to enlarge, the futures to be feparated, and he never afterwards retained the faculty of fucking to fuch a degree as formerly. From that time, his head had always a tendency to fall to the left fide, and it could not be kept erect without a fupport.— After he had recovered from the fever, an eruption appeared on his right fhoulder, and on the left fide of his breaft, and began to fpread by degrees over his body, his brows, and the back of his head. The matter difcharged by the *fores* had a faltifh, difagreeable fmell, a watery appearance, and feemed to be of fo infectious infectious a nature, as to raife painful and fimilar *fores* on the arms and breaft of his mother. He was eafier, and he generally flept better, when the *fores* or *puftules* difcharged much.

Thus, I have given as diftinct an account of the fymptoms of this extraordinary cafe, prior to the time I was called, as I could learn from the mother and the midwife.—I was fent for to fee him when he was about feven weeks old; at that time, his eyes were a little turned to the left fide, and feemingly fixed in that fituation; they were always open, even when he flept, yet, they were not more prominent than ufual; but the pupils were confiderably dilated: light did not in any wife affect them. He was fo acute in hearing, that the leaft noife ftartled or wakened him. His mouth was very large, and conftantly open, except when he fwallowed any thing. His tongue was fhort, and far back in his mouth: he could never be faid to be altogether free from fever, for his pulfe continued fo quick and irregular, that I could not number it. There was fcarcely any alteration on him from the feventh to the tenth week of his age, except that he became weaker, and his head ftill larger: he took his bread and milk as he ufed to do. About the end of the tenth week he began to be a little convulfed, on the left fide in particular: his left arm was turned about in fuch a manner, as that the palm of the hand was uppermoft; and the joints of the leg and thigh on the fame fide, were fometimes fo ftiff, that they could not be *moved.* There were fpafms over his whole body, which went off, and returned alternately. He remained in this ftate till the thirteenth week; and after that, entirely loft the faculty of fucking; but his mother fupplied that defect, by often milking her breafts into his mouth. In this fituation he lived five weeks, then death put a period to his miferable exiftence. Before he died he was much reduced, fo ftiff and cold, that, had it not been from his breathing, and attempting to cry, and fometimes a writhing of his body, none could have believed that he was alive: It is fcarcely poffible to communicate any adequate idea of fuch a wretched object. His head, when expofed to the light, had almoft, in every direction, a tranfparent appearance, fuch as a lantern has when covered with oiled paper. During the whole courfe of the difeafe, he was perfectly regular in his bowels, and his ftools were of the natural colour; but within a week of his death they became fœtid, and his belly hard; and a day or two before he died, he made more water than he ufed to do. He continued to take nearly his ufual quantity of food till the night of his death, and even that night, took two or three fpoonsful of it. He always drank a great deal; flept little or none, but cried, or *fretted conftantly.*

Having

Having obtained leave from his parents to open his head, I called in my neighbour, Dr. Napier, who had ſeen the child when alive, and likewiſe a young gentleman in the town, to ſee it performed. As I wiſhed to preſerve the water carefully, till meaſured, I drew it off with a trocar and canula: after I had drawn off all I could in that manner, I made a longitudinal inciſion in the direction of the ſagittal ſuture, firſt through the ſkin and teguments, and next through the dura mater; and between the dura and pia mater, (where, no doubt, the whole of the water had been lodged) I found a little more, which I likewiſe took out. There were, in all, ſixteen gills of water in the head, and as pure as if taken from a ſpring: the bones of the head were flexible, and ſo ſoft, that I could puncture them with a lancet almoſt in any place. At firſt ſight the ſkull appeared like a ſhell, and not a veſtige of brain to be ſeen, or at leaſt, nothing but empty membranes; but on diſſecting the whole of the membranes, and dividing the medulla oblongata, I found a part of the two poſterior lobes remaining, which were of a ſolid membranous ſubſtance. The left lobe was a little thicker than the right, and of a greeniſh blue. When I cut into it, I found the inſide almoſt of the ſame colour; the right had more of a grey colour. The thickeſt part of them did not exceed three-quarters of an inch, or an inch at moſt. Of the fore part of the brain, nothing remained but a ſhrivelled membrane. All the reſt of the parts had their natural colour. When the membranes, and what remained of the brain, were diſſected, and the veſſels divided that ſupply the brain, &c. there was ſcarcely a drop of blood to be ſeen; and only about ʒj. or ʒij. of bloody water. Although the head had been ſeparated from the body, and immerſed for a conſiderable time in water, it could not be whiter, nor freer of blood than it was. I cannot conceive how the child could live ſo long, with his brain in ſuch a ſtate, and ſuch a preſſure of water upon it; perhaps, a much leſs quantity of water in the ventricles of the brain, would have proved fatal ſooner.

Might not much crying, without a prediſpoſition to the diſeaſe, be the exciting cauſe of hydrocephalus in children? Might not the frequent cleanſing of the ſtomach and the bowels by *purges*, during the firſt three or fonr weeks of this child's age, have prevented or carried off the diſeaſe?

I am ſorry that the ſhortneſs of the time allowed for the operation, did not permit me to inveſtigate the brain with ſuch exactneſs, as to be able to give you a more diſtinct account of it; but from what I have ſaid, you can ſee that the caſe is a very remarkable one. When you have a leiſure moment, I

ſhall hope to have the pleaſure of your opinion concerning it.

I am, with much reſpect,

Dear Sir,

Your's, &c.

